# Can rectal catheters be avoided during paediatric urodynamic studies?

**DOI:** 10.1080/2090598X.2019.1668176

**Published:** 2019-09-25

**Authors:** Abhilash Cheriyan, Arun Jacob Philip George, Antony Devasia, J. Chandrasingh

**Affiliations:** Department of Urology, Christian Medical College and Hospital, Vellore, India

**Keywords:** Catheter, neurogenic bladder, urodynamics, paediatric urology, pressure–flow study

## Abstract

**Objective**: To determine if the interpretation of urodynamic studies (UDS) in children without a rectal catheter may be similar to multi-channel studies, as UDS in children are challenging and can sometimes be difficult to interpret.

**Patients and methods**: In this retrospective pilot study, 115 paediatric pressure–flow studies were included. A blinded investigator was given two sets of UDS traces. The first set had the vesical trace of all children and the second set had the multi-channel trace. The agreement between the interpretations of both the sets was tested by Cohen’s κ, and sensitivity, specificity, and predictive values were expressed with 95% confidence intervals (CIs). The voiding pattern was compared and Pearson’s correlation coefficient was used to analyse the pressure at maximum urinary flow (Q_max_).

**Results**: The most common indications for UDS were neurogenic bladder and posterior urethral valves. The interpretation of compliance and detrusor overactivity by single-channel analysis had a positive predictive value of 92.1% (95% CI 84.7–96.1%) and 89.4% (95% CI 78.3–95.6%), respectively, and a negative predictive value of 100% and 97.1% (95% CI 89.5–99.2%) respectively, in comparison to multi-channel analysis. Children with underactive detrusor were identified reliably by analysing the straining pressure pattern and flow curve. Amongst children who voided, the pressure at Q_max_ showed a moderate correlation (Pearson’s coefficient = 0.53) between the two groups.

**Conclusion**: Rectal catheters may be avoided in a carefully selected group of children undergoing UDS who only need filling phase assessment.

**Abbreviations:** DO: detrusor overactivity; EBC: expected bladder capacity; P_abd_: abdominal pressure; P_det_: detrusor pressure; PUV: posterior urethral valve; (N)(P)PV: (negative) (positive) predictive value; P_ves_: vesical pressure; Q_max_: maximum urinary flow rate; UDS: urodynamic studies; UI: urinary incontinence

## Introduction

Urodynamic studies (UDS) in children have a well-established role in the management of complex urological and neuro-vesical pathologies [1,2]. Multi-channel cystometry is currently the ‘gold standard’ for urodynamic evaluation [3]. Although the placement of a rectal catheter in addition to a vesical catheter enables subtraction of abdominal pressure (P_abd_) from vesical pressure (P_ves_), it is sometimes counterproductive in children. Distress caused during the procedure often makes them uncooperative. The traces thus obtained are hard to interpret due to excessive artefacts. In addition, the incremental benefit of measuring the P_abd_ may be of questionable value in certain groups of patients where the only indication for this procedure is to assess bladder compliance and detrusor overactivity (DO) to decide on fluid intake, the frequency of clean-intermittent catheterisation, and anticholinergic medications.

Based on these observations in routine practice, the present study aimed to determine if pressure–flow studies in children can be interpreted satisfactorily with single-channel cystometry to avoid the discomfort of a rectal catheter. There are few studies that have evaluated the role of one-channel cystometry [4,5] in identifying bladder overactivity and stress urinary incontinence (UI) in adults. Ricci et al. [6] found single-channel cystometry to be a useful adjunct to clinical examination in women with UI. Single-channel cystometry interpreted carefully in relation to clinical findings is considered a reasonably accurate, safe and cheap method for diagnosing neurogenic bladder dysfunction, especially in spinal cord injuries [7]. In another study comparing single- and multi-channel cystometry as a screening tool for detrusor instability in women, single-channel studies had acceptable specificity but the predictive value was poor as a screening tool [8]. There are no such studies in the paediatric population to our knowledge.

## Patients and methods

### Study population and design

**Figure 1.**
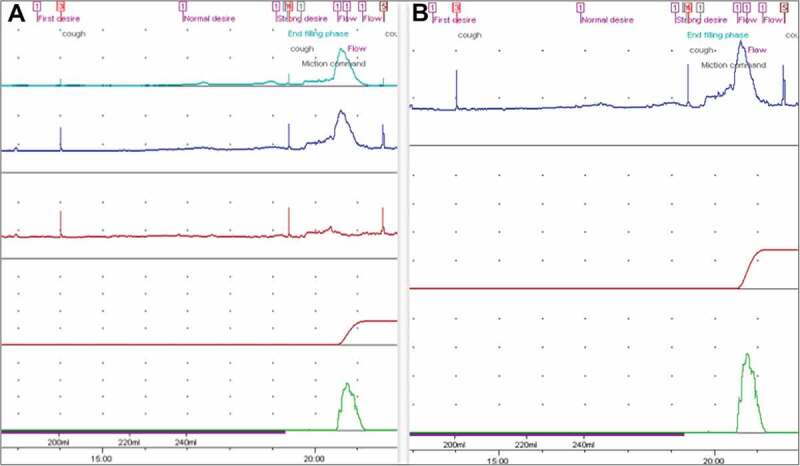
An example of two sets of urodynamic trace for the same patient. (A) Multi-channel trace with P_det_ (light blue), P_ves_ (blue), P_abd_ (red), flow (green). (B) Single-channel P_ves_ trace obtained by removing P_abd._

A retrospective cohort study was conducted, which included all children who underwent UDS at our centre during the period July 2014 to June 2017. In all, 118 successfully completed UDS were conducted in this period and 115 were included in this study. Three children had two UDSs during this period and only the first study was included in these cases. UDSs of children aged >1 year up to 16 years were included, which is considered the upper age-limit for paediatric patients at the authors’ centre. Incomplete studies and patients that lacked relevant clinical data were omitted. Data were acquired from the hospital’s electronic database. The single-channel traces were obtained by digital subtraction of the P_abd_ and P_ves_ traces from the multi-channel trace (Figure 1). Patient identifiers were removed and only relevant clinical data were provided. Traces were interpreted by a blinded paediatric urologist with >10-years’ experience working in the same unit. Single-channel traces were first assigned for interpretation and the findings were recorded. The same investigator was then assigned the multi-channel traces for interpretation. The two sets of interpretations were then compared.

### UDS

All children had a detailed history taken, examination, and completed a 48-h bladder diary. Ultrasonography of the abdomen was used for upper tract assessment, uroflowmetry, and renal function tests. All children had undergone multi-channel cystometry by standard institutional protocols based on the International Children’s Continence Society guidelines [9]. Studies were done in the sitting position if the child was able, and all studies were done in the presence of the parents with informed written consent. No drugs or sedatives were used during the study. Patients were asked to come for the test after emptying their bowel if possible, or the study was re-scheduled for the next day after administrating a laxative the previous night.

Filling and simultaneous measurement of P_ves_ were done by a 6-F double-lumen transurethral catheter placed in the bladder. Lignocaine jelly (0.2%) was used to aid the introduction of the catheter. P_abd_ was measured simultaneously with a small rectal balloon catheter. Normal saline solution (0.9%) at room temperature [10] was instilled in a retrograde manner, with physiological filling rate calculated as weight (in kg) divided by 4. At the beginning of the filling phase and at regular intervals, the child was instructed to cough to ensure that the abdominal and vesical pressure lines were balanced, when possible [11].

Detrusor pressure (P_det_) was calculated by subtracting P_abd_ from P_ves_. The P_abd_ and P_ves_ traces were digitally masked to obtain a trace with only the P_ves_ for comparison. Voiding command was given at normal desire to void in older children. In non-toilet-trained children, signs of discomfort or whenever the child voided was considered as the end-filling phase.

### Outcome measurement

Comparisons between the interpretation of multi-channel UDS and of the P_ves_ trace alone were made with respect to bladder compliance, detrusor instability, and voiding pressures.

Poor compliance in a multi-channel trace was defined as bladder pressure at the expected bladder capacity (EBC) of >10 cmH_2_O. Similarly, an increase in P_ves_ (∆P_ves_) by ≥10 cmH_2_O at EBC for age was the threshold to detect poor compliance in the single-channel trace [12]. In children where EBC was not reached during the filling phase, pressure at end-filling was used to determine the compliance. The EBC for age was determined by the equation derived by Koff [13], which is important to take into consideration, as unlike adults, compliance changes with age in children. DO was reported when there were involuntary provoked or unprovoked contractions with an increase in pressure by 15 cmH_2_O during the filling phase from the baseline [14].

The voiding phase was analysed by:

1. The pattern of flow curves and
2. Comparing pressures at maximum urinary flow (Q_max_).

In children who strained to void, the blinded investigator compared the pattern of the pressure and flow curves to identify if the child was straining to void, as absolute pressure measurements were not possible. Children who could not void, or voided <50% of the EBC [15], were also excluded from analysis of pressures during voiding. In others, the P_det_ at Q_max_ (pressure at Q_max_ on conventional pressure–flow studies) and ∆P_ves_ at Q_max_ (the corresponding P_ves_, after accounting for the baseline P_ves_ at the start of filling phase) were compared.

### Statistical methods

Data entry was done using EpiData Entry Client (EpiData Association, Odense, Denmark). Statistical analysis was done using the Statistical Package for the Social Sciences (SPSS®), version 20 (SPSS Inc., IBM Corp., Armonk, NY, USA). Categorical data were analysed using descriptive statistics and reported as frequency and percentage. Compliance and DO derived from both methods in the study were represented by 2 × 2 contingency tables and Cohen’s κ value with 95% CI was calculated to assess the agreement between the two methods [16]. Sensitivity, specificity, positive predictive value (PPV) and negative PV (NPV) were calculated for single-channel cystometry considering multi-channel as the ‘gold standard’. The correlation between the voiding pressures (P_det_ at Q_max_ and ∆P_ves_ at Q_max_) were analysed by calculating the Pearson’s coefficients.

## Results

### Demography

In all, 115 paediatric UDS were reviewed, which included 86 boys and 29 girls (Table 1). The median (interquartile range) age of the children was 12 (8–15) years. Neurogenic bladder was the commonest indication for UDS (56 children), followed by posterior urethral valves (PUVs; 41 children). Children with anatomical abnormalities and dysfunctional voiding were few in number, with 14 and four, respectively, in each group.

**Table 1.** Demographic data.

Variable	Value
Gender distribution, *n* (%)	
Boys	86 (74.8)
Girls	29 (25.2)
Age, years, median (interquartile range)	12 (8–15)
Indication for UDS, *n* (%)	
Neurogenic bladder	56 (48.6)
PUV	41 (35.6)
Anatomical abnormalities	14 (12.2)
Dysfunctional voiding	4 (3.5)

### Filling phase

In Table 2, the number of children with normal and decreased compliance by both methods of interpretation is shown. Interpretation of compliance was concordant in 109 of the 115 children (94.7%), with a sensitivity of 100% (95% CI 94.9–100%) and specificity of 86.7% (95% CI 73.2–94.95%). The PPV was 92.1% (95% CI 84.7–96.1%) and NPV was 100%. The agreement between the two methods of interpretation was very good; the κ value was 0.89 (95% CI 0.80–0.98).

**Table 2.** Comparison of interpretation of compliance and DO between single- and multi-channel studies.

		Compliance (multi-channel cystometry), *n*	
		Decreased	Normal
Compliance (single-channel cystometry), *n*	Decreased	70	6
	Normal	0	39
	Total	70	45
		DO (multi-channel cystometry), *n*	
		Overactive	Normal
DO (single-channel cystometry), *n*	Overactive	42	5
	Normal	2	66
	Total	44	71

The next parameter compared was DO. Table 2 shows the comparison of DO as interpreted by a single- and multi-channel trace. Results of the blinded interpretations were concordant in 94% (108/115 children), with a sensitivity of 95.5% (95% CI 84.5–99.4%) and specificity of 93% (95% CI 84.3–97.7%). The PPV of the single-channel trace was 89.4% (95% CI 78.3–95.6%) and NPV was 97.1% (95% CI 89.5–99.2%). The κ value, which represents the agreement between the two methods, was 0.87 (95% CI 0.78–0.96).

### Voiding phase

#### Voiding pattern analysis

In all cases, the flow curve in conjunction with the pressure curves could reliably predict if the child was straining to void. In children who had a straining pattern whilst voiding (*n *= 55; Table 3), although actual voiding pressures could not be compared, the blinded investigator could identify straining pattern of flow and pressure curves suggestive of an underactive detrusor. This was done reliably with single-channel cystometry in all patients.

**Table 3.** Breakdown of children in each category who were included for analysis of voiding pressures.

	Excluded due to low voided volume and low cystometric capacity (*n* = 19)	Excluded due to straining to void (*n* = 55)	Voiding phase analysed by comparing the pressure at Q_max_ (*n* = 41)
Neurogenic bladder (*n* = 56)	11	45	0
PUV (*n* = 41)	3	3	35
Anatomical abnormalities (*n* = 14)	5	5	4
Voiding dysfunction (*n* = 4)	0	2	2

#### Voiding pressure analysis

The voiding phase pressures were analysed in a total of 41 children of which 35 had PUVs. Correlation between P_det_ at Q_max_ [mean (SD) was 50.36 (20.24) cmH_2_O] and ∆P_ves_ at Q_max_ [mean (SD) was 58.36 (21.07) cmH_2_O] was acquired by calculating the Pearson’s coefficient, the value of which was 0.53 (*P* < 0.05). In all, 19 children were excluded due to either low voided volume or low bladder capacity. Another 55 were excluded as they strained to void, which made accurate voiding phase pressure measurement impossible in these children.

## Discussion

Pressure–flow studies are challenging and time-consuming in children. Trained personnel and a friendly environment are prerequisites for studying bladder function in children [1,17]. Although invasive urodynamic evaluation with multi-channel cystometry gives an accurate assessment of pressures to assess lower urinary tract dysfunction (LUTD), it can cause a great deal of discomfort to children resulting in artefacts. Although there is no scale to measure the degree of physical and emotional distress, it has been well documented in adults [18].

It is important to be aware of the various causes of artefacts that may be present in a paediatric UDS. Most children are either anxious or restless during the study. Straining, change in position, movement of the tubes, rectal contractions, and faulty calibration can lead to artefacts [19]. DO may be precipitated by irritation caused by the catheter, cold saline, supra-physiological filling rate, and coughing. Additionally, the placement of catheters in a narrow urethra may cause difficulty in voiding. Bladder sensation was not studied, as it is subjective and relevant only in older, toilet-trained children.

In the present study, the Cohen’s κ was used to compare both tests instead of McNemar’s test, due to the small number of observations in two of the cells in the contingency table (Table 2) making it unsuitable for our analysis. There was very good agreement between single- and multi-channel cystometry for diagnosing poor compliance with a PPV and NPV of >92%. Likewise, there was very good agreement between single- and multi-channel traces with respect to DO. Five children were over-diagnosed with DO by the single-channel trace due to intermittent rectal contractions during the study.

The interpretation of the filling phase of the study did not change, irrespective of the availability of the P_abd_ trace in most of the patients. Single-channel interpretation had a high NPV of 100% and 97% for the diagnosis of poor compliance and DO, respectively. Six children were over-diagnosed as having poor compliance, while five were over-diagnosed with DO by single-channel interpretation. False positives were equally distributed in the neurogenic bladder and PUV groups. False positive DO on single-channel interpretation corresponded to an increase in P_abd_. The benefit of avoiding the discomfort of a rectal catheter vs a small chance of over-diagnosis of poor compliance and DO is debatable.

Comparison of the voiding phase between the two methods of interpretation is primarily based on: (i) the flow and pressure curve pattern and (ii) voiding pressures. Children with neurogenic bladders were excluded from the analysis of voiding pressure. Children with neurogenic bladder are often on intermittent clean catheterisation and the usual indications to do a pressure–flow study in these patients are to determine upper tract safety, the need for bladder drainage and anticholinergic medication. Although accurate voiding phase pressure measurements are not possible without measuring P_abd_, in children with underactive and decompensated bladders, the staccato or interrupted pattern of flow, along with careful interpretation of the P_ves_ trace could reliably identify abdominal straining, thus enabling clinical decision-making.

In children with non-neurogenic LUTD, as mentioned earlier, the interpretation of the filling phase was concordant in most cases. However, the correlation of voiding pressures, by comparing absolute values of P_det_ and ∆P_ves_ at Q_max_ was only moderate. This subgroup included children with dysfunctional voiding as well as children with sequelae of PUV fulguration. As the correlation is at best, moderate, pressure–flow studies without the P_abd_ trace for clinical scenarios that require meticulous assessment of the voiding phase are not recommended by the authors.

There is no literature available with regard to single-channel UDS in children. In the present study, the single-channel traces were obtained from the multi-channel traces that were available for the children. This negated any other possible confounders influencing the pressure traces. Comparison of a single- and multi-channel study done at two different times is not comparable due to multiple other confounders, especially in children. At the same time, a derived single-channel trace may be a source of bias, as these traces were balanced with the help of a rectal catheter at the time of multi-channel study. This may have resulted in a falsely accurate interpretation of the derived single-channel graph.

Another important limitation of the present study is the retrospective nature of the study. Secondly, the relatively small sample size may not be sufficient to establish the non-inferiority of single-channel cystometry. Additional blinded independent reviewers would help in determining inter-observer consistency in interpretation. Most children included in the present study had neurogenic bladder or PUVs and the results may not be generalisable across all diagnoses. One may also argue that most children with a neurogenic bladder are insensate and may not be bothered by a rectal catheter. However, the authors believe that the emotional and psychological aspects cannot be ignored in children, and it is of value if the rectal catheter can be avoided in some.

Although the present study had its limitations, these results should encourage larger prospective studies to be undertaken to conclusively address this hypothesis, because often in our pursuit for accurate diagnoses the discomfort and trauma caused by these investigations are often over-looked, especially in children. The ideal study design would be to do the single- and multi-channel study consecutively in the same sitting and compare the same with a *t*-test.

## Conclusion

This retrospective blinded analysis suggests that rectal catheters may be avoided in a carefully selected group of children requiring assessment of only the storage phase.
